# Supplementary material for: Resolving the full spectrum of human genome variation using Linked-Reads
Source: Genome Res. 2019 Apr;29(4):635–45. doi: 10.1101/gr.234443.118 (PMC6442396; doi:10.1101/gr.234443.118)
Supplement: Supplemental Material [file supp_gr.234443.118_Supplemental_longranger-2.2.zip › longranger-2.2.tar.bz2!/lr2.2-public/lib/stan/cmdstan/stan/doc/api/html/agrad_2rev_2matrix_2variance_8hpp_source.html]

Stan: src/stan/agrad/rev/matrix/variance.hpp Source File


|  |  |
| --- | --- |
|  | Stan  2.5.0  probability, sampling & optimization |


- Main Page
- Related Pages
- Namespaces
- Classes
- Files

- File List
- File Members

All Classes Namespaces Files Functions Variables Typedefs Enumerations Enumerator Friends Macros Pages

- src
- stan
- agrad
- rev
- matrix

variance.hpp

Go to the documentation of this file.

1 #ifndef STAN\_\_AGRAD\_\_REV\_\_MATRIX\_\_VARIANCE\_HPP

2 #define STAN\_\_AGRAD\_\_REV\_\_MATRIX\_\_VARIANCE\_HPP

3

4 #include <vector>

5 #include <boost/math/tools/promotion.hpp>

6 #include <stan/math/matrix/Eigen.hpp>

7 #include <stan/math/matrix/mean.hpp>

8 #include <stan/agrad/rev/var.hpp>

9 #include <stan/agrad/rev/vari.hpp>

10 #include <stan/agrad/rev/matrix/stored\_gradient\_vari.hpp>

11 #include <stan/math/error\_handling/matrix/check\_nonzero\_size.hpp>

12

13 namespace stan {

14

15  namespace agrad {

16

17  namespace { // anonymous

18

19  var calc\_variance(size\_t size,

20  const var\* dtrs) {

21  vari\*\* varis = (vari\*\*) memalloc\_.alloc(size \* sizeof(vari\*));

22  for (size\_t i = 0; i < size; ++i)

23  varis[i] = dtrs[i].vi\_;

24  double sum = 0.0;

25  for (size\_t i = 0; i < size; ++i)

26  sum += dtrs[i].vi\_->val\_;

27  double mean = sum / size;

28  double sum\_of\_squares = 0;

29  for (size\_t i = 0; i < size; ++i) {

30  double diff = dtrs[i].vi\_->val\_ - mean;

31  sum\_of\_squares += diff \* diff;

32  }

33  double variance = sum\_of\_squares / (size - 1);

34  double\* partials = (double\*) memalloc\_.alloc(size \* sizeof(double));

35  double two\_over\_size\_m1 = 2 / (size - 1);

36  for (size\_t i = 0; i < size; ++i)

37  partials[i] = two\_over\_size\_m1 \* (dtrs[i].vi\_->val\_ - mean);

38  return var(new stored\_gradient\_vari(variance, size,

39  varis, partials));

40  }

41

42  }

43

51  var variance(const std::vector<var>& v) {

52  stan::math::check\_nonzero\_size("variance(%1%)",v,"v",(double\*)0);

53  if (v.size() == 1) return 0;

54  return calc\_variance(v.size(), &v[0]);

55  }

56

57  /\*

58  \* Return the sample variance of the specified vector, row vector,

59  \* or matrix. Raise domain error if size is not greater than

60  \* zero.

61  \*

62  \* @tparam R number of rows

63  \* @tparam C number of columns

64  \* @param[in] m input matrix

65  \* @return sample variance of specified matrix

66  \*/

67  template <int R, int C>

68  var variance(const Eigen::Matrix<var,R,C>& m) {

69  stan::math::check\_nonzero\_size("variance(%1%)",m,"m",(double\*)0);

70  if (m.size() == 1) return 0;

71  return calc\_variance(m.size(), &m(0));

72  }

73

74  }

75 }

76

77 #endif

vari.hpp

stan::agrad::memalloc\_

memory::stack\_alloc memalloc\_

**Definition:** var\_stack.cpp:16

stan::agrad::sum

fvar< T > sum(const Eigen::Matrix< fvar< T >, R, C > &m)

**Definition:** sum.hpp:14

mean.hpp

stored\_gradient\_vari.hpp

var.hpp

stan::math::mean

boost::math::tools::promote\_args< T >::type mean(const std::vector< T > &v)

Returns the sample mean (i.e., average) of the coefficients in the specified standard vector...

**Definition:** mean.hpp:23

Eigen.hpp

stan::memory::stack\_alloc::alloc

void \* alloc(size\_t len)

Return a newly allocated block of memory of the appropriate size managed by the stack allocator...

**Definition:** stack\_alloc.hpp:168

stan::agrad::var

Independent (input) and dependent (output) variables for gradients.

**Definition:** var.hpp:27

stan::math::size

int size(const std::vector< T > &x)

**Definition:** size.hpp:11

stan::agrad::variance

var variance(const std::vector< var > &v)

Return the sample variance of the specified standard vector.

**Definition:** variance.hpp:51

check\_nonzero\_size.hpp

stan::math::check\_nonzero\_size

bool check\_nonzero\_size(const char \*function, const T\_y &y, const char \*name, T\_result \*result)

Return true if the specified matrix/vector is of non-zero size.

**Definition:** check\_nonzero\_size.hpp:33


---

[ Stan Home Page ]

*© 2011–2014,
Stan Development Team.*
